# Hospital Meetings, &c.

**Published:** 1897-04-03

**Authors:** 


### HOSPITAL MEETINGS, &C.

### LONDON HOMŒOPATHIC HOSPITAL.

The annual meeting of the London Homœopathic Hospital was held in the board-room on Friday, 26th ult., Lord Emlyn (treasurer) in the chair. The report stated that during 1896, the first complete year during which the new hospital has been occupied, the in-patients had numbered 1,031 and the out-patients 14,511, both slightly in excess of the estimates made by the board, and there seemed to be every probability of these figures being exceeded in 1897. During the year 196 persons had been sent to the Convalescent Home at Eastbourne. The ordinary income amounted to £6,208, legacies, £1,618, and donations for the endowment of beds, £2,000. The ordinary expenditure amounted to £8,377, and the extraordinary expenditure to £377. The board called attention to the fact that the receipts from private nursing showed a further decrease from £1,039 in 1895 to £575 in 1896.—The Chairman, in moving the adoption of the report, said that the total deficit at the end of 1896 was £4,225.—This sum had been met out of capital, but the management was very anxious to replace the amount so used, and therefore appealed to the supporters of the hospital for this amount, and also for a further £5,000 to complete the new building fund.—Captain Cundy seconded, and the report was adopted.—Votes of thanks to the honorary officers were passed, the retiring members of the board of management, the auditors, and the medical staff were re-elected, and the election of the new members of the board was confirmed. The meeting closed with a vote of thanks to the chairman.

### ROYAL LONDON OPHTHALMIC HOSPITAL.

The annual meeting of the governors of the Royal London Ophthalmic Hospital was held at the hospital on Tuesday, 30th ult., Sir John Lubbock, Bart., M.P., in the chair. The Chairman moved the adoption of the report, which stated that the number of patients treated during 1896 was 28,080, viz., 2,006 in-patients and 26,074 out-patients. During the year 823 cases were refused treatment, as their circumstances were not such as to entitle them to gratuitous relief. The income amounted to £4,809, an increase of £105 on 1895, and the expenditure was £7,771, an increase of £272. Mr. H. P. Sturgis seconded, and the report was adopted. Votes of thanks and the re-election of officers completed the business of the meeting.

### ORPHAN WORKING SCHOOL.

Mr. Horace B. Marshall presided over the anniversary festival of the Orphan Working School, held at the Hotel Metropole on Tuesday, 30th ult., when about 300 sat down to dinner, including the Archdeacon of London, Lady Agnes Anderson, Mr. Alderman Treloar, Colonel Wellesley Robinson, Mr. Alderman Pound, Sir Clarence Smith, Mr. Basil Woodd Smith (treasurer). Mr. Alderman Treloar, in giving "The Army and Navy," expressed the satisfaction it was to them all to see that the chairman was following in the footsteps of his revered father. (Applause.) The Chairman, in proposing "Success to the Orphan Working School," said it was no new institution, for, for 139 years it had carried on a great and good work, and during that time nearly 6,000 boys and girls had been maintained, educated, and fitted for the battle of life within its walls. There were two branches—the senior branch at Haverstock Hill, and the junior branch at Hornsey Rise; and, in addition, they had a convalescent home at Margate. The children were received, trained, and educated, and at 14 were started in life. In the last 25 years nearly 50 boys had been received into his firm, and he could say that they had almost without exception turned out well. Having read the reports of the Government inspectors on the recent examinations, the Chairman said they required at least £13,000 a year to maintain the schools in an efficient condition, and he was extremely desirous of raising a fourth of that sum that night. Only £25 a year was required to keep a child, and he trusted that night he would have an extremely good subscription list. It was a record year, and they had a record attendance, but it was also a record year for appeals. Still he thought that year they could not do better than assist such a work as that which relieved the widow and the fatherless. (Applause.) The Secretary (Mr. A. Coote) read the subscription list, which included 250 guineas from the Chairman, and totalled about £2,740.

